# Analyses of Physical and Psychological Characteristics of “Squid Game” Characters Using East Asian Biopsychosocial Personality Theories and Body Mass Index

**DOI:** 10.3390/bs14100907

**Published:** 2024-10-08

**Authors:** Seokyung So, Danilo Garcia, Jeongyun Lee, Ji Hwan Kim, Sang Yun Han, Soo Jin Lee, Han Chae

**Affiliations:** 1 School of Korean Medicine, Pusan National University, Busan 50610, Republic of Korea; sarah9293@naver.com; 2 Department of Social Studies, University of Stavanger, 4021 Stavanger, Norway; danilo.garcia@icloud.com; 3 Lab for Biopsychosocial Personality Research (BPS-PR), International Network for Well-Being; 4 Promotion of Health and Innovation Lab (PHI), International Network for Well-Being; 5 Department of Behavioral Sciences and Learning, Linkoping University, 581 83 Linköping, Sweden; 6 Centre for Ethics, Law and Mental Health (CELAM), University of Gothenburg, 405 30 Gothenburg, Sweden; 7 Department of Psychology, University of Gothenburg, 405 30 Gothenburg, Sweden; 8 Department of Sasang Constitutional Medicine, Division of Clinical Medicine 4, School of Korean Medicine, Pusan National University, Yangsan 50612, Republic of Korea; leejyun@pusan.ac.kr (J.L.); jani77@pusan.ac.kr (J.H.K.); 9 College of Korean Medicine, Daejeon University, Daejeon 34520, Republic of Korea; drhan@dju.kr; 10 Department of Psychology, Kyungsung University, Busan 48434, Republic of Korea

**Keywords:** body mass index, drama character, Eum-Yang (Yin-Yang), perceived biopsychological characteristics, Sasang Personality Questionnaire (SPQ), Sasang typology, Squid Game

## Abstract

Media characters’ physical and psychological traits are crucial for character development and audience engagement. This study examines East Asian perspectives on the audience’s perceptions of the physical appearance and personality, using Eum-Yang biopsychological Sasang theory, of five characters from the Netflix series “Squid Game”. A total of 221 university students assessed the traits of five “Squid Game” characters using the Sasang Personality Questionnaire (SPQ) and a visual Body Mass Index (BMI). ANOVA and Profile Analysis revealed significant and comprehensive differences in the SPQ and its subscales (behavior, SPQ-B; cognition, SPQ-C; and emotion, SPQ-E) as well as BMI among the five drama characters. More specifically, Seong Gi-hun (SGH) and Han Mi-nyeo (HMN) were So-Yang types (high SPQ, moderate BMI), Cho Sang-woo (CSW) and Kang Sae-byeok (KSB) were So-Eum types (low SPQ, low BMI), and Jang Deok-su (JDS) was Tae-Eum type (moderate SPQ, high BMI). Psychological profiling showed two patterns: V-shaped (high SPQ-B, low SPQ-C, high SPQ-E) for SGH, HMN, and JDS, and A-shaped (low SPQ-B, high SPQ-C, low SPQ-E) for CSW and KSB. These results contribute to media psychology by highlighting the relevance of Eum-Yang and Sasang typology for creating and analyzing complex characters, thereby enhancing global understanding for East Asian biopsychosocial theories.

## 1. Introduction

Television dramas offer a rich tapestry of complex characters, each with distinct physical appearances, psychological traits, and social traits. These attributes are essential not only for character development but also to engage the audience’s emotional connection; that is, to create a parasocial relationship to the characters [1,2,3,4,5,6]. While the Western perspective has predominantly influenced character analysis through various psychological and physiological frameworks, there is a notable absence of studies employing East Asian perspectives and understanding of biopsychosocial characteristics, such as the Eum-Yang (Yin-Yang) theory and Sasang typology [7,8,9,10].

The Eum-Yang theory has been used in all East Asian disciplines, including philosophy, psychology, biology, and medicine [9,11,12]. The Eum (Yin) generally represents femininity, introversion, inhibition, passivity, coldness, closure, and darkness, while Yang signifies masculinity, extraversion, activation, openness, activity, heat, and light [9]. Recently, the Eum-Yang theory has been reinterpreted as describing the dualistic nature of biomedical phenomena and used to understand dynamic equilibrium, interdependence, and complementarity within complex adaptive biopsychological systems [8,9,13,14]. These two opposite, yet closely tied, characteristics are conceptualized as inhibited (Eum) or activated (Yang) biopsychological states that can be operationalized using a reliable and valid clinical tool, namely the Sasang Personality Questionnaire (SPQ) [9,15,16,17,18]. The SPQ (Table 1) provides three subscales, of behavioral attitudes (SPQ-B), cognitive styles (SPQ-C), and emotional responses (SPQ-E), along with a total score (SPQ-T). A high SPQ-T score indicates a Yang state characterized by extraversion, sociability, flexibility in cognition, quick movement, and high emotionality, while a low SPQ-T score indicates a Eum state characterized by introversion, stability, organization, caution, and minimal behavioral and emotional changes [9,19]. In other words, the SPQ is a practical tool to understand television drama characters’ drives and approaches and avoidance motivations in the form of emotions, cognitions, and behaviors [1,14,20,21].

The Sasang typology is a traditional Korean personalized medical typology based on an understanding of human biopsychological nature within the East Asian Eum-Yang theory [16]. It explains the unique psychophysiological characteristics of four Sasang types (Tae-Yang, So-Yang, Tae-Eum, So-Eum). It also suggests methods for disease prevention, treatment, and rehabilitation using acupuncture and medical herbs specific to each type [22]. Each Sasang type has unique psychophysiological characteristics based on the individual’s SPQ-T and Body Mass Index (BMI; a clinical indicator of bodily development and obesity calculated by dividing weight by the square of height and often used as a perceived physical characteristic) [16,22,23]. For example, So-Yang individuals report high SPQ-T scores, thus are characterized as extroverts with a muscular body; while So-Eum individuals report low SPQ-T score, thus are introverts with a relatively lean and small body [15,16]. Tae-Eum individuals, on the other hand, are characterized by a developed and large body, as measured by the ponderal index (PI) and BMI [24], with psychological characteristics in between the So-Yang and So-Eum types [18,22]. Interestingly, research shows that the biopsychological characteristics of three Sasang types (i.e., So-Yang, So-Eum, and Tae-Eum) are similar to those of the Choleric, Melancholic, and Phlegmatic humoral types described by Hippocrates and Galen, the Athletic, Asthenic, and Pyknic physique types described by Kretschmer, the Mesomorph, Ectomorph, and Endomorph somatotypes described by Sheldon, and the Pitta, Vata, and Kapha doshas of Prakriti within Indian Ayurveda [22]. Thus, the Sasang typology is valid and acknowledged across different cultures.

### 1.1. The Perceived Physical and Psychological Characteristics of Media Characters

Previous studies have reported the successful analysis of biopsychological characteristics of fictional characters in children’s animation [1,8] and adult first-person shooter (FPS) games [20] using the SPQ and BMI, thus extending the usefulness of the SPQ and BMI beyond the clinical diagnosis of Sasang types [25]. For example, when applied to characters in the animation “Pororo the Little Penguin”, Pororo (a mood-making penguin with curiosity and a sense of discovery) and Petty (an outgoing and merry girl penguin who is good at sports) were found to be typical So-Yang types, while Loopy (a feminine and shy girl beaver who is good at cooking) was a So-Eum type, and Poby (a polar bear with the biggest body and a gentle nature) was confirmed as a Tae-Eum type [1]. Similarly, fictional characters in the game Overwatch, Tracer (a time-jumping pistol-wielding adventurer with irrepressible energy) was a So-Yang type, Mei (a former climatologist using ice-based powers) was a So-Eum type, and Zarya (a former weight-lifting world champion using a particle-based cannon) was confirmed as a Tae-Eum type [20].

Moreover, previous studies have repeatedly reported that BMI reflects unique bodily characteristics of Sasang type groups [15,25]. So-Yang types exhibited characteristics of a large and long body shape accompanied by a high Resting Metabolic Rate, a high PI, a low percentage of skeletal muscle, and good digestive function. In contrast, So-Eum types showed a slender and lean body shape, with a lower BMI and PI, low Resting Metabolic Rate, high percentage of skeletal muscle, and low digestive function [22,24,26]. And the Tae-Eum types were found to have physical characteristics between those of So-Yang and So-Eum types [26].

While these and other previous studies have successfully demonstrated that the East Asian Eum-Yang (Yin-Yang) biopsychological theory can be applied to analyze media characters [8,16,21,27], characters in games or animations have been criticized for lacking realism due to their simplistic narratives and limited interactions among virtual or unrealistic characters (cf. [28,29,30]). Therefore, this study aims to analyze the perceived biopsychological features of drama characters in a renowned episodic drama [31,32], namely “Squid Game” (2021), using the methodology of previous studies among characters in games and animations [1,8,16,20,25]. After all, television dramas present more diverse and complex characters that are more vividly portrayed by real actors and provide an ideal case for the current study [6,33,34,35].

### 1.2. The Present Study

“Squid Game” (2021) is a South Korean suspense thriller that achieved unprecedented success on Netflix, receiving one Golden Globe Award and six Primetime Emmy Awards [31,32]. The storyline revolves around 456 people in dire socioeconomical situations who are invited to participate in a secret survival contest with a prize money of KRW 456 billion (around USD 39 million). The participants play six Korean traditional children’s games but with life-and-death stakes. The individuals portrayed in the “Squid Game” managed to garner empathy [31,32] and form parasocial relationships among viewers with diverse cultural backgrounds [36].

**Figure 1:**
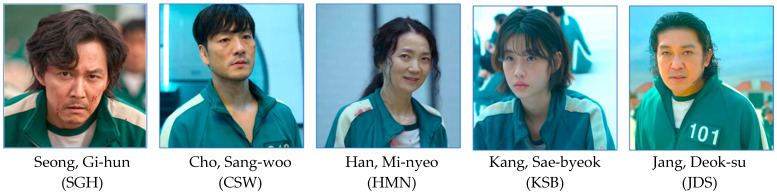
Profile pictures of the five main characters of the “Squid Game” discussed in the current study (character images reprinted/adapted with permission from © Netflix Studios, LLC., Los Gatos, CA, USA).

In this study, three male and two female characters of “Squid Game” were utilized based on their screen time, story significance, and their distinctive physical and psychological characteristics (Figure 1). The male characters chosen were the gambler Seong Gi-hun (SGH, participant #456), the investment fund manager Cho Sang-woo (CSW, #218), and the gangster Jang Deok-su (JDS, #101), while the female characters were the North Korean defector Kang Sae-byeok (KSB, #67) and the swindler Han Mi-nyeo (HMN, #212) [31]. We argue that the global popularity of the series and the distinct physical and psychological profiles of its characters make it a compelling subject for our aim and analysis [32].

By focusing on these five prominent drama characters from “Squid Game”, this study aims to determine if assessments of physical and biopsychosocial personality and Sasang types, as reported by Korean university students using SPQ and BMI, can objectively and coherently validate these character’s attributes and typology. If so, this research will bridge the gap between audience perception and scientific validation of characteristics in television drama characters. Moreover, by employing BMI and biopsychosocial measures, this study might demonstrate the applicability and universality of these constructs in a global entertainment context [1,8,20,21,27] and contribute to advance media psychology by enriching the understanding of East Asian biopsychosocial theories in contemporary settings.

## 2. Materials and Methods

### 2.1. Participants and Procedures

This study was conducted among 221 university students in the Busan metropolitan area who had previously watched the drama “Squid Game” and who voluntarily chose to participate. The data collection was conducted from 9 December 2022 to 27 December 2022 after receiving approval from the Pusan National University Institutional Review Board (PNU IRB/2022_121_HR) in accordance with the Declaration of Helsinki.

To facilitate recall of the five characters biopsychological characteristics, participants were at first shown video clips featuring each character: Seong Gi-hun (https://youtu.be/3723jCnjE4g (accessed on 26 December 2022), 3:08~4:10), Cho Sang-woo (https://youtu.be/LWumOK6_Y6Y (accessed on 26 December 2022), 0:35~1:50), Han Mi-nyeo (https://youtu.be/W_OozSyUips (accessed on 26 December 2022), 0:15~1:25), Kang Sae-byeok (https://youtu.be/z88IiUMcaCE (accessed on 26 December 2022), 6:10~7:20), and Jang Deok-su (https://youtu.be/cwb4YF9xMj8 (accessed on 26 December 2022), 0:00~1:10) [31]. Subsequently, participants were asked to provide the perceived physical and psychological features of the five drama characters using SPQ and BMI.

We also collected the participants’ viewership of “Squid Game” (0 episodes, 1–4 episodes, 5–8 episodes, all 9 episodes) and familiarity with each character (Don’t know at all, Don’t know well, Average, Know, Know very well). Participants who had not watched the drama (0 episodes) were excluded from the study. The perceived BMI and SPQ of the drama characters, along with familiarity with characters, gender, and age of participants who watched all 9 episodes, were used for analysis.

### 2.2. “Squid Game” and the Five Characters

“Squid Game” (2021), consisting of 9 episodes, is a Korean suspense thriller directed by Hwang Dong-hyuk and has achieved the highest success on Netflix’s video streaming platform as of now [31]. It has become Netflix’s most-viewed series, capturing the top spot in 94 countries, reaching over 142 million member-households and 1.65 billion viewing hours within its initial four weeks of release.

The series tells the story of a survival game with a prize of USD 39 million, inviting 456 people in dire straits to participate. The drama depicts the story of “social losers” based on the director’s experiences of economic hardships and class disparities. Using Korean children’s folk games (Red Light, Green Light, Sugar Honeycombs, Tug-of-War, Playing Marbles, Glass Stepping Stones, and Squid Game) and addressing contemporary Korean issues, the drama successfully elicited empathy from audiences of various cultural backgrounds [31,32,37].

In this study, five drama characters from “Squid game” [31] with similar age ranges and distinct physical and psychological characteristics were selected based on their screen time and story importance (Figure 1). The male gambler Seong Gi-hun (SGH, participant #456), who survives to win the top prize, is emotionally sensitive, burdened by debt, yet considerate of the weak. Meanwhile, the male investment fund manager Cho Sang-woo (CSW, #218), childhood friend of SGH, survives to second place thanks to his cold and calculated demeanor [31]. Kang Sae-byeok (KSB, #67), a female North Korean defector, survives to third place due to her calm and composed demeanor in each game. Han Mi-nyeo (HMN, #212), a female swindler, reaches joint sixth place thanks to her emotional and opportunistic behavior, employing various tricks and methods. Jang Deok-su (JDS, #101), a male gangster, with his large physique, adeptness in physical combat, and lack of inhibition towards murder, shares sixth place due to a joint fall caused by HMN.

### 2.3. Measures

#### 2.3.1. Sasang Personality Questionnaire (SPQ)

The SPQ is a multidimensional psychological test based on Eum-Yang (Yin-Yang) theory and Confucianism, consisting of 20 items (4-point scale) that measure temperament or biopsychological characteristics [9,17].

It comprises three subscales of behavioral attitudes (SPQ-B), cognitive styles (SPQ-C), and emotional reactions (SPQ-E), as well as a total score (SPQ-T) (Table 1). SPQ-B measures the degree of extraversion, sociability, cooperativeness, activeness, diligence, and vitality in behavioral attitudes, while SPQ-C assesses independence, flexibility, confidence, and straightforwardness in cognitive styles, and SPQ-E measures empathy, emotional sensitivity, emotional intensity, passion, and anxiety [9,14]. Scores on each subscale reflect unique psychological characteristics, and individuals may exhibit adaptive or maladaptive behaviors based on their attitudes towards perceiving and responding to stressful situations [19,38] (Table 1).

Based on the SPQ-T (i.e., combining all three subscales), individuals can be categorized into three Sasang types. So-Yang individuals (top 30%) exhibit activated biopsychological characteristics, such as being extroverted, sociable, optimistic, quick to react, easily excitable, and emotionally responsive; So-Eum individuals (bottom 30%) possess inhibited biopsychological traits, such as introversion, caution, passivity, consistency, organization, quietness, and stability; and Tae-Eum individuals (middle 40%) are in between So-Yang and So-Eum types, showing no specific tendency in responsiveness to external stimuli [15,39].

Previous studies have reported the following internal consistency (Cronbach’s alpha) in adults: SPQ-T = 0.704, SPQ-B = 0.861, SPQ-C = 0.685, and SPQ-E = 0.709. Test–retest reliabilities with five-month intervals in high school students were SPQ-T = 0.763, SPQ-B = 0.766, SPQ-C = 0.727, and SPQ-E = 0.704 [9,15].

#### 2.3.2. Body Mass Index (BMI)

**Figure 2:**
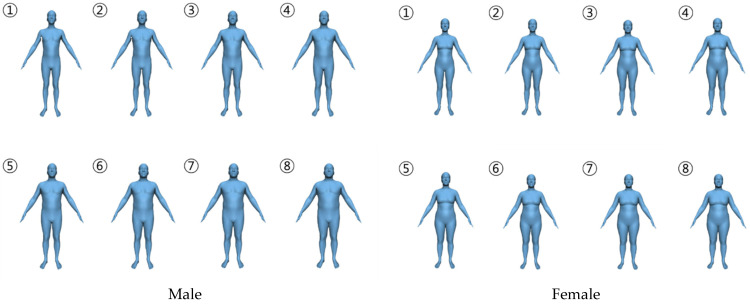
Representative BMI model of male (**left**) and female (**right**) characters used for determining body features from 17.5 to 38.5 with a 3-point interval.

This study presented illustrative images with BMIs ranging from 17.5 to 38.5 at intervals of 3 for males and females (Figure 2). Participants were asked to select the most similar one for each drama character. The images used were created using the BMI visualizer (https://bmi.is.tue.mpg.de/ (accessed on 26 December 2022)) [1,8,20].

### 2.4. Statistical Analysis

For demographic characteristics, the χ^2^ test was used to analyze participants’ familiarity with the drama characters based on the number of episodes watched and gender. A *t*-test was used to analyze age differences between genders.

The differences in physical (BMI) and psychological (SPQ-T) characteristics among the five drama characters, assessed by the participants, were analyzed using Analysis of Variance (ANOVA). Levene’s test was employed to assess the homogeneity of variance, and Welch’s test or the Games–Howell test was used for post hoc analysis.

The significant differences in biopsychological profiles (SPQ-B, SPQ-C, and SPQ-E) among the five drama characters were analyzed using Profile Analysis and ANOVA. In Profile Analysis, Greenhouse–Geisser correction was applied when Mauchly’s W was significant, and both Flatness and Parallelism were provided. Levene’s test was used in ANOVA to assess homogeneity of variance, and Welch’s test or the Games–Howell test was employed for post hoc analysis.

We used Jamovi 2.4.14 (The jamovi project, https://www.jamovi.org (accessed on 20 February 2024)) for all statistical analysis. The results were presented as mean ± standard error or frequency (%). Statistical significance levels were set at *p* < 0.05, *p* < 0.01, and *p* < 0.001.

## 3. Results

### 3.1. Demographic Features of Male and Female Viewers

Among the total of 221 participants, data from 195 participants who watched all nine episodes (Season 1) were used for the analyses in this study (Table S1). While no significant differences were found in familiarity with drama characters between genders (Table S2), males (24.4 ± 2.92) and females (21.8 ± 2.22) differed significantly (t = 7.09, *p* < 0.001) with regard to age.

### 3.2. Physical and Psychological Features of the “Squid Game” Characters

**Figure 3:**
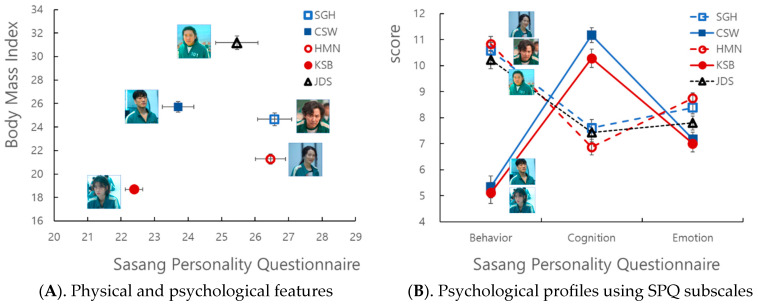
Scores in physical (Body Mass Index) and psychological (Sasang Personality Questionnaire) features of the five “Squid Game” characters as rated by university students in the present study (character images reprinted/adapted with permission from © Netflix Studios, LLC. 2021). SGH: Seong, Gi-hun, CSW: Cho, Sang-woo, Han, HMN: Mi-nyeo, Kang, KSB: Sae-byeok, and JDS: Jang, Deok-su.

The perceived psychological and physical characteristics of the five drama characters as rated by Korean university students are presented in Figure 3A. The psychological characteristics, measured with the SPQ-T scores, were: SGH = 26.58 ± 3.65, HMN = 26.46 ± 3.18, JDS = 25.46 ± 4.50, CSW = 23.70 ± 3.76, and KSB = 22.39 ± 4.67. The ANOVA revealed a significant difference among the five drama characters (F = 39.912, *p* < 0.001). The post hoc analysis showed that the drama characters rated as high SPQ-T scorers were SGH, HMN, JDS, and those rated as low scorers were CSW and KSB. The physical characteristics, measured with BMI, were: JDS = 31.18 ± 4.21, CSW = 25.72 ± 3.31, SGH = 24.67 ± 3.83, HMN = 21.29 ± 3.11, and KSB = 18.70 ± 1.86. The ANOVA revealed a significant difference among the five drama characters (F = 470.379, *p* < 0.001) and the post hoc analysis showed significant differences (*p* < 0.05) between all five drama characters.

Considering these results (Figure 3A), the “Squid Game” characters could be divided based on three distinct biopsychological characteristics: SGH and HMN with high SPQ-T and moderate BMI, CSW and KSB with low SPQ-T and low BMI, and JDS with medium SPQ-T and high BMI. That is, according to the biopsychological features of each Sasang type, SGH and HMN could be associated with the So-Yang type characterized by high SPQ-T and moderate BMI, JDS with the Tae-Eum type characterized by high BMI, and CSW and KSB with the So-Eum type characterized by low SPQ-T and low BMI.

### 3.3. Psychological Profile of the “Squid Game” Characters Using SPQ Subscales

Two contrasting psychological profiles were identified based on the three SPQ subscale scores (Figure 3B): a V-shaped profile (high SPQ-B, low SPQ-C, high SPQ-E) for SGH, HMN, and JDS, and an A-shaped profile (low SPQ-B, high SPQ-C, low SPQ-E) for CSW and KSB.

Profile Analysis based on the SPQ subscales was used to examine differences in psychological profiles among drama characters. The Greenhouse–Geisser correction was used since Mauchly’s W was significant (W = 0.698, *p* < 0.001). Significant differences in psychological profile were observed between drama characters (Flatness with Greenhouse–Geisser correction, df = 1.536, F = 37.215, *p* < 0.001; Parallelism with Greenhouse–Geisser correction, df = 6.143, F = 231.030, *p* < 0.001), indicating that the psychological profiles of drama characters differed significantly.

The ANOVA revealed significant differences between drama characters in each SPQ subscale score (Figure 3B). Regarding SPQ-B, there was a significant difference among drama characters (F = 149.441, *p* < 0.001) and the post hoc tests showed that HMN (10.84 ± 2.01), SGH (10.58 ± 2.26), and JDS (10.23 ± 2.5) were rated as having high SPQ-B scores compared to CSW (5.34 ± 2.96) and KSB (5.11 ± 2.90). Significant differences were found in SPQ-C (F = 35.633, *p* < 0.001), with post hoc tests showing significant differences among CSW (11.17 ± 2.04), KSB (10.28 ± 2.53), SGH (7.61 ± 2.23), and HMN (6.87 ± 2.16). JDS (7.43 ± 2.53) did not differ significantly from SGH and HMN in SPQ-C, but was rated as significantly lower compared to CSW and higher compared to KSB. Significant differences were also found between drama characters in SPQ-E (F = 39.912, *p* < 0.001) with post hoc tests showing significant differences among HMN (8.75 ± 1.47), SGH (8.39 ± 1.71), JDS (7.80 ± 1.90), CSW (7.18 ± 1.83), and KSB (7.00 ± 2.20).

In sum (Figure 3A,B), the complex biopsychological features of each drama character can be systematically presented using the SPQ subscales of the East Asian biopsychological Sasang taxonomy.

## 4. Discussion

In this study, we aimed to bridge the gap between audience perception and scientific validation of characteristics in television drama characters in a global entertainment context with the application of an East Asian biopsychosocial theory [1,8,20,21,27]. In short, the perceived psychological traits and physical appearances of the five drama characters in “Squid Game” were analyzed, and their distinctive Sasang types (Figure 3A) and comprehensive psychosocial profiles (Figure 3B) were successfully presented by employing SPQ and BMI [31,32].

Firstly, the five drama characters can be objectively categorized into three types of Sasang typology using SPQ and BMI (Figure 3A), which describes detailed and extensive psychological and physical attributes [9,15,19,39]. More specifically, SGH and HMN could be associated with the So-Yang type, characterized by high SPQ-T and moderate BMI, which manifests as activated biopsychological characteristics such as extroversion, sociability, flexibility in cognition, quick reactions, excitability, and emotional responsiveness. CSW and KSB could be associated with the So-Eum type, characterized by low SPQ-T and low BMI, represents inhibited biopsychological traits such as introversion, caution, passivity, consistency, organization, quietness, and stability, along with a slender and lean body shape [15,22,24,25,26]. Additionally, JDS was found to be the only Tae-Eum type, characterized by mid-range SPQ-T and high BMI, representing a large and long body shape with psychological characteristics in between So-Yang and So-Eum types.

Among the So-Yang types, the emotional SGH emphasizes an adaptive aspect of a gambler, responding positively to stressful situations, and showing an active demeanor by caring for marginalized individuals in each game. In contrast, the maladaptive aspect of swindler was emphasized with HMN, who interprets and reacts negatively to stress, exhibiting exaggerated behavior and voices, extreme emotional responses, impulsivity, and irrational actions. Among the So-Eum types, the adaptive aspect of a North Korean defector is emphasized in KSB, who approaches each game rationally, calmly, and steadily, while the maladaptive aspect of an investment fund manager is highlighted in CSW, who is calculated, self-centered, and cold-hearted, betraying colleagues emotionlessly for victory. JDS, as a Tae-Eum type gangster, frequently displays the maladaptive aspect of a gang leader, utilizing his large physique to fight and monopolize distributed food [31].

Secondly, the psychological profiles of the five drama characters were illustrated in two composite profiles using three SPQ subscales, SPQ-B, SPQ-C, and SPQ-E, (Figure 3B). This enables a more realistic, vivid, and complex portrayal of the drama characters compared to the flat, shallow, and superficial features of fictional characters seen in animations or games, allowing viewers to empathize with the drama characters and making the story of the drama more engaging [3,40].

Drama characters such as SGH and HMN of the So-Yang type, as well as JDS of the Tae-Eum type, exhibited a V-shaped profile based on the SPQ sub-scales (high SPQ-B, low SPQ-C, high SPQ-E) in Figure 3B. They are generally in an activated biopsychological state of Yang, exhibiting extroversion, sociability, quick reactions, and excitability in behavior and emotions, but their cognitive styles are rather meticulous, showing traits of being detail-oriented, prone to fussing over small matters, being pessimistic, or being rigid (Table 1) [9,15,31]. On the other hand, CSW and KSB, classified as So-Eum types, displayed an A-shaped profile (low SPQ-B, high SPQ-C, low SPQ-E) as shown in Figure 3B. They are generally in an inhibited biopsychological state of Eum, exhibiting introversion, stability, logic, and distant characteristics in behavior and emotions, but their cognitive styles are the opposite, showing traits of being self-centered, adaptable to situations, intuitive, or cool-headed.

Indeed, the results of the current study revealed the applicability and generalizability of East Asian biopsychological theory, Eum-Yang and Sasang typology, in understanding and validating the attributes of drama characters in a global entertainment context. It is in line with previous studies showing that popular virtual characters in children’s animations [1,8] and first-person shooter games [20] can be categorized into Sasang types. These unique psychological characteristics of Sasang types manifest themselves in adaptive or maladaptive ways in real-life situations under stress (Table 1).

Protagonists typically exhibit positive, adaptive, and mature traits, displaying humanity and compassion by helping others, which allows the audience to empathize with and identify with them. In contrast, characters who oppose or must be overcome by the protagonist tend to display negative, maladaptive, and immature traits, often resorting to betrayal or manipulation for personal gain [41,42]. The contrast in characteristics, behaviors, and narrative roles between adaptive characters such as Seong Gi-hun (SGH) and Kang Sae-byeok (KSB), and maladaptive characters such as Cho Sang-woo (CSW) and Han Mi-nyeo (HMN), helps create tension as they face extreme moral dilemmas and make drastic choices in their fight for survival [38,43]. This dynamic not only provides compelling storylines intertwined with drama and a sense of reality that immerses the audience, but also lends coherence to the drama’s plot [19,31,44]. Moreover, it allows the audience to gain insight into the diverse aspects of human nature and the psychological changes that emerge under extreme circumstances.

To sum up, this study empirically demonstrates that SPQ and BMI with clinical validity and efficacy [25] can be utilized not only in analyzing fictional characters of animations [1,21] and games [20], but also in the systematic illustration of the biopsychological characteristics of tangible drama actors within complex real-life scenarios. Subsequently, Eum-Yang and Sasang typology using SPQ and BMI might be useful for creating noble and immersive drama characters.

### Limitations

Firstly, since this study was conducted with Korean university students and a Korean drama, there is a need to verify this with participants from other cultures, nationalities, ages, genders, occupations, and television dramas from, for example, India, Anglo-America, the Middle East, Europe, and the African region [10,33,34]. However, considering that the methodology used in this study was successfully applied in children’s animations of Korea [1,21] and FPS games made in the United States [20], similar results are likely to be observed [27].

Secondly, this study illustrated multidimensional V-shaped and A-shaped psychological profiles and subsequently exhibited their adaptive or maladaptive psychological features, which are determined by one’s attitudes toward perceiving and responding to stressful situations [19]. An empirical examination is needed to determine how adding psychological maturity (Table 1) to the description of drama characters affects the level of audience engagement and emotional empathy [38].

Thirdly, there is a need to analyze factors influencing the perceived physical and psychological characteristics of actors [12,45,46]. For example, actors often change their body shape, weight, beard, hairstyle, and clothing for new roles, alter their image, switch genres, introduce method acting, or opt for villainous roles, and pursue changes in the character’s occupation, socio-economic status, and more. Thus, it is necessary to analyze in detail the factors that influence the perceived characteristics of actors in relation to the characters they play in, for example, a television series [47].

## 5. Conclusions

This study showed that the perceived characteristics of five television drama characters can be successfully categorized into distinct Sasang types based on their SPQ and BMI scores, revealing coherent and detailed psychological and physical attributes. This typology probably enables a more realistic and vivid portrayal of the characters’ biopsychosocial features, enhancing the audience’s ability to relate with them and making the drama’s narrative more engaging. This study not only contributes to the field of media psychology (see among others [48,49,50]) by empirically demonstrating the robust nature of East Asian biopsychosocial theories and their potential utility in various media formats for character analysis and efficient character design. More importantly, the results also enhance the understanding of the Eum-Yang and Sasang typology in a global entertainment context [8]. Furthermore, it may also be beneficial to students and professionals with an interest in East Asian traditional medicine and area studies [1,20,21,27]. Indeed, the study’s methodology offers a valuable pedagogical tool for teaching students how to evaluate physical and psychological characteristics to gain practical insights into the application of biopsychosocial measures, enhancing their observational and analytical skills. This approach not only enriches the educational experience but also fosters a deeper understanding of East Asian biopsychosocial theories [14].

## Figures and Tables

**Table 1:** Typical adaptive and maladaptive features of low and high Sasang Personality Questionnaire (SPQ) subscale score profiles.

SPQ Subscales		Adaptive	Maladaptive
SPQ-Behavior	High	Active, energetic, proactive, expressive, extroverted	Hyperactive, impulsive, aggressive, easily affected
	Low	Stable, calm, disciplined, focused, introverted	Passive, avoidant, withdrawn, tired, unable to adapt
SPQ-Cognition	High	Flexible thinker, optimistic, intuitive, cool-headed	Self-centered, haphazard, moody, uncontrollable
	Low	Systematic, consistent, meticulous, fair, reasonable	Cranky, pessimistic, rigid, ruminative
SPQ-Emotion	High	Passionate, engaging, warm, intimate, emotional	Unstable, irrational, hostile, hypersensitive, dependent
	Low	Rational, stable, calm, distant, logical	Cold, dispassionate, indifferent, isolated, emotionless

## Data Availability

The datasets used and/or analyzed during the current study are available from the corresponding author on reasonable request.

## Supplementary Materials

The following supporting information can be downloaded at: https://www.mdpi.com/article/10.3390/bs14100907/s1, Table S1: Number of episodes watched according to the gender, Table S2: Familiarity with characters according to the gender.
