# Supplementary material for: Analyses of Physical and Psychological Characteristics of “Squid Game” Characters Using East Asian Biopsychosocial Personality Theories and Body Mass Index
Source: Behav Sci (Basel). 2024 Oct 8;14(10):907. doi: 10.3390/bs14100907 (PMC11504730; doi:10.3390/bs14100907)
Supplement: Supplementary file 1 [file behavsci-14-00907-s001.zip › behavsci-3165025-supplementary.pdf]

Table S1. Number of episodes watched according to the gender

	Male (n=90)	Female (n=131)	Total (n=221)	Statistical analysis
1~4 episodes	9 (64.3)	5 (35.7)	14 (100)	χ^2=4.49, p=0.106
5~8 episodes	3 (25.0)	9 (75.0)	12 (100)	
all 9 episodes	78 (40.0)	117 (60.0)	195 (100)	

Table S2. Familiarity with characters according to the gender

	Male (n=78)	Female (n=117)	Total (n=195)	Statistical analysis
Seong, Gi-hun (SGH)				
Unfamiliar				χ^2=2.95, p=0.228
Moderate	15 (51.7)	14 (48.3)	29 (100)	
Familiar	44 (40.7)	64 (59.3)	108 (100)	
Very familiar	19 (32.8)	39 (67.2)	58 (100)	
Cho, Sang-woo (CSW)				
Unfamiliar	2 (100.0)	0 (0.0)	2 (100)	χ^2=4.61, p=0.203
Moderate	16 (48.5)	17 (51.5)	33 (100)	
Familiar	52 (38.2)	84 (61.8)	136 (100)	
Very familiar	8 (33.3)	16 (66.7)	24 (100)	
Kang, Sae-byeok (KSB)				
Unfamiliar	2 (66.7)	1 (33.3)	3 (100)	χ^2=7.32, p=0.062
Moderate	22 (56.4)	17 (43.6)	39 (100)	
Familiar	43 (33.9)	84 (66.1)	127 (100)	
Very familiar	11 (42.3)	15 (57.7)	26 (100)	
Jang, Deok-su (JDS)				
Unfamiliar	2 (100.0)	0 (0.0)	2 (100)	χ^2=7.76, p=0.051
Moderate	19 (55.9)	15 (44.1)	34 (100)	
Familiar	45 (35.4)	82 (64.6)	127 (100)	
Very familiar	12 (37.5)	20 (62.5)	32 (100)	
Han, Mi-nyeo (HMN)				
Unfamiliar	4 (80.0)	1 (20.0)	5 (100)	χ^2=6.61, p=0.086
Moderate	23 (50.0)	23 (50.0)	46 (100)	
Familiar	40 (34.8)	75 (65.2)	115 (100)	
Very familiar	11 (37.9)	18 (62.1)	29 (100)	
